# Metformin increases the expression of proinflammatory cytokines and inhibits supraspinatus fatty infiltration

**DOI:** 10.1186/s13018-023-04163-z

**Published:** 2023-09-12

**Authors:** Jong Pil Yoon, Sung-Jin Park, Dong-Hyun Kim, Seok Won Chung

**Affiliations:** 1 Department of Orthopaedic Surgery, School of Medicine, Kyungpook National University, Daegu, Korea; 2 Department of Orthopaedic Surgery, School of Medicine, Konkuk University Medical Center, Seoul, Korea

**Keywords:** Metformin, Inflammatory response, Fatty infiltration, Supraspinatus atrophy, Histology, Rotator cuff tear

## Abstract

**Background:**

After a rotator cuff (RC) tendon tear, the supraspinatus (SS) inflammatory response induces fatty infiltration (FI). Metformin has the effect of regulating the initial inflammatory response of atrophic muscles. Therefore, this study aimed to investigate the effect of metformin use on modulating the expression of proinflammatory cytokines and SS FI in an acute RC tear rat model.

**Methods:**

This study used 26 male Sprague–Dawley rats. Animals were randomly divided into two groups: The metformin group received metformin for 5 days after cutting the RC tendon, and the control group was administered only with saline after cutting the tendon. Metformin 50 mg/kg was intraperitoneally injected for 5 days. Three rats in each group were sacrificed 5 days after SS tendon rupture surgery, and 10 rats in each group were sacrificed 14 days after surgery. The SS was sampled 5 days after SS tendon tear surgery, and the expression of proinflammatory cytokines was measured by quantitative reverse-transcription polymerase chain reaction (qRT-PCR). On day 14 after sampling, histological analysis of the SS was performed using hematoxylin and eosin, Masson’s trichrome, and picrosirius red staining.

**Results:**

On day 5 of surgery, the expression values of interferon gamma (increased 7.2-fold, *P* < .01), tumor necrosis factor alpha (increased 13-fold, *P* < .05), interleukin-1β (increased 4.7-fold, *P* < .001), and interleukin-6 (increased 4.6-fold, *P* < .01) increased significantly in the metformin group compared with those in the control group. As a result of Oil Red O staining, SS FI was significantly suppressed in the metformin group compared with that in the control group (metformin group, 305 ± 50.3 µm^2^, *P* < .001; control group, 3136 ± 662.8 µm^2^, *P* < .001). In addition, the SS volume of the metformin group was not reduced compared with those of the control group, and the morphology and structure of the SS were better preserved.

**Conclusions:**

The results of this study revealed that metformin can increase the expression of proinflammatory cytokines and suppress SS fat infiltration in delayed sutures.

## Background

RC tear is one of the most common shoulder disorders in humans and incurs enormous medical costs to patients, severe pain in the shoulder joint, muscle weakness, and limited mobility [1]. Despite successful surgical intervention, RC tears do not heal spontaneously, and poor surgical prognosis and high re-tear rates remain a problem [2–5]. Factors that adversely affect improved healing following RC tendon suture include RC muscle fiber atrophy and fat accumulation in and around the muscle fibers. In these RC tears, FI is an irreversible, muscle degenerative change that results in muscle weakness, shoulder dysfunction, limited range of motion, and impaired tendon–bone healing [6, 7].

Massive RC tears induce an inflammatory response in muscles and can have detrimental effects on the development of chronic diseases, leading to muscle atrophy and FI of the skeletal muscles [8–11]. Previous studies have suggested that the RC muscle becomes inflamed in the presence of an RC tendon tear [12] and that this acute inflammation plays a detrimental role in the development of chronic muscle diseases [7, 13]. In an actual animal experiment, the number of inflammatory cells such as neutrophils and monocytes/macrophages peaked at the first 5–7 days in the SS muscles with ruptured full-thickness RC tendons, and significant changes were noted in proinflammatory and anti-inflammatory cytokines [5, 12, 14, 15]. Clinical studies have also reported muscle changes and overall changes in cytokine levels, metalloprotease levels, and growth factors after RC tendon rupture [16]. This muscle inflammation after an RC tendon rupture affects the degree of FI and subcellular changes in the skeletal muscles [5, 17]. Therefore, the modulation of muscle inflammation in RC tears may be an effective target to improve RC tendon–bone healing [16].

Metformin is an FDA-approved drug for hypoglycemic diabetes that has been used to treat type 2 diabetes mellitus since 1957 [18–20]. It does not induce hypoglycemia, has an excellent antihyperglycemic effect, reduces cost, and has excellent pharmacological properties as a first-line drug, such as having fewer side effects [19–23]. In addition to its antihyperglycemic effects, metformin has the potential to influence proinflammatory cytokine levels by activating macrophages within the inflammatory response [24]. Elevated proinflammatory cytokine levels can trigger the apoptosis of fibro-adipogenic progenitors (FAPs) and the activation of satellite cells, consequently mitigating the fatty degeneration of FAPs [24, 25]. The effects of metformin on the inflammatory response of the SS muscle resulting from an RC tear have not been established. Our hypothesis was that the metformin-induced activation of immune cells during the initial stages of RC rupture might have the potential to suppress SS FI.

This study aimed to verify the inhibitory effect of metformin on FI in an acute RC tear rat model. In this study, an RC tendon tear model was created, and the expression of cytokines associated with the early postoperative acute inflammatory response was evaluated. FI of the SS muscle was evaluated 14 days after RC tendon cutting, and muscle atrophy was confirmed by histological staining.

## Methods

### Animal model

All animal testing procedures were approved by the Institutional Animal Committee designated by the author’s institution. This study used 9-week-old male Sprague–Dawley rats, which were raised in a specific pathogen-free facility. The temperature of the breeding facility was 22 ± 2 °C, and the light–dark cycle was adjusted to 12 h/12 h, and food and water were given freely. Based on previous study (comparison between 2 groups: mean difference 436 µm^2^, standard deviation 96 µm^2^, *α* error = 0.05, *b* error = 0.2, 2-tailed comparison), power analysis determined that 5 specimens were needed per group to detect a significant difference in a quantitative analysis of FI [26]. A total of 26 rats were randomly divided into the metformin group and the control group. Three rats each of the control and metformin groups were sacrificed for qRT-PCR on day 5. After 14 days, the left shoulders of 5 rats were used for the Oil Red O staining, and the right shoulders of 10 rats were used for the histological analysis.

### Surgical procedures

**Fig. 1:**
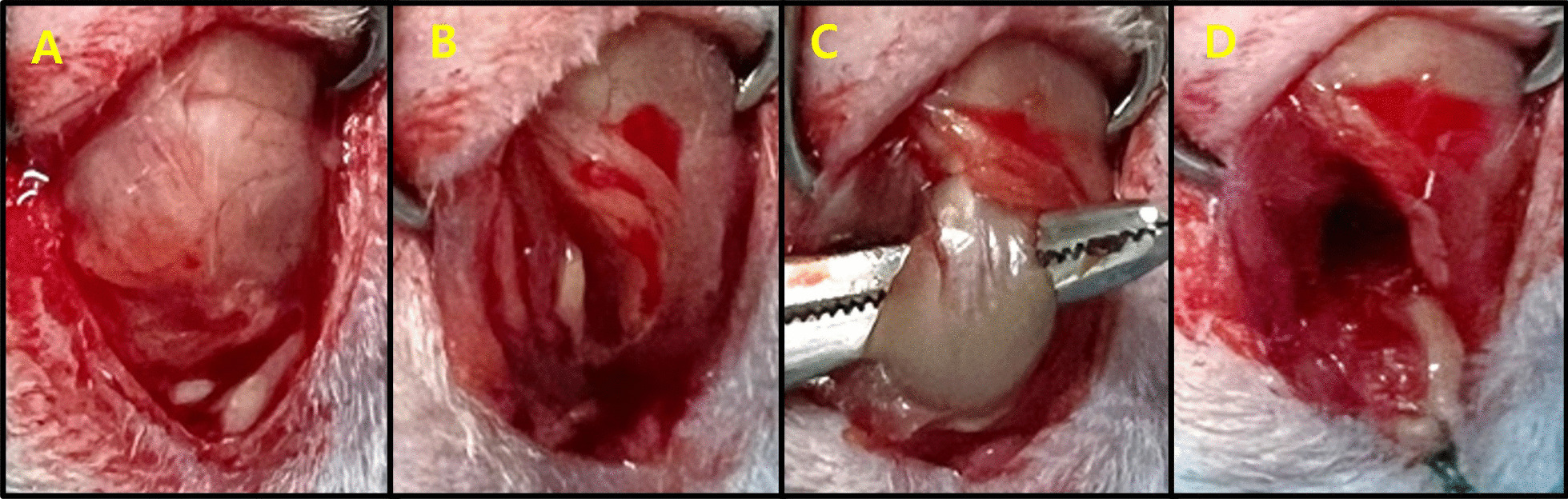
Animal model and surgical procedure. **A** A longitudinal skin incision of 3–4 cm was made along the scapula, and the deltoid muscle was exposed. **B** A longitudinal 2-cm incision was made in the exposed deltoid muscle. **C** The SS tendon was identified on the inside of the deltoid muscle and marked with a suture. **D** The SS tendon was incised at the greater tuberosity using a knife

**Fig. 2:**
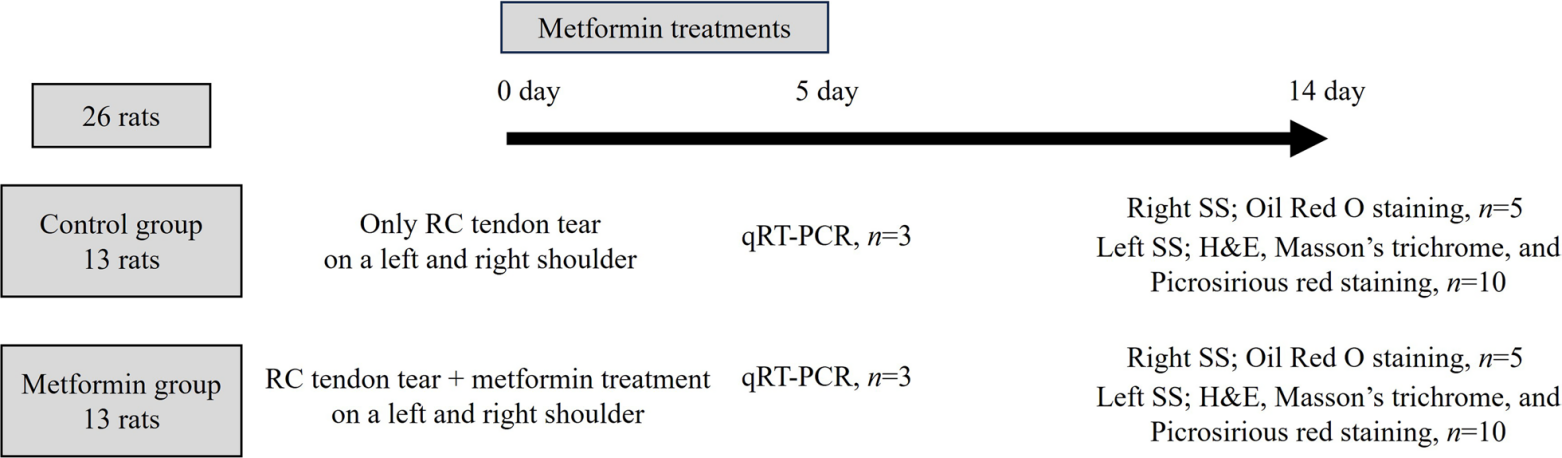
Experimental timeline schematic for animal experiments. A total of 26 rats were randomly allocated into two groups, each consisting of 13 rats. RC tendon tear surgery was performed on both shoulders of all rats. The metformin group received drug administration for a duration of 5 days. Additionally, all rats were sacrificed 14 days post-surgery. Oil Red O staining was conducted on the five SS extracted from the left shoulder, while histological evaluation was carried out on the ten SS from the right shoulder

Anesthesia was induced with 5% isoflurane delivered by oxygen at 4 L/min via inhalation in an induction chamber, and anesthesia was maintained with 2% isoflurane via a nose cone. The fur on the shoulder was moderately shaved to expose the skin and sterilized to maintain sterility. An RC tear procedure was then performed [1]. Briefly, the scapula was palpated, and a 3-cm long incision was made. As a result, the deltoid muscle was split, and the SS tendon was exposed (Fig. 1A). The SS tendon was separated using the Metzenbaum, and the tendon was cut at the tendon–bone site using a knife blade (Fig. 1B). The SS where the tendon was cut was marked by tying a knot using Ethibond (Ethicon, Somerville, New Jersey, USA) (Fig. 1C). The metformin group was intraperitoneally injected with metformin after surgery. Metformin (Hanmi Pharm, Suwon, Korea) was prepared at a dose of 50 mg/kg/day and was injected intraperitoneally each time for 5 days. Drug doses and methods were referenced in a previous study [18]. Moreover, 5 and 14 days after surgery, all mice were sacrificed, and the SS was harvested (Fig. 2).

### qRT-PCR

According to the manufacturer’s protocol, total RNA was isolated from rat SS tissue using NucleoSpin RNA Plus kit (Macherey–Nagel, Duren, Germany) and then reversed into cDNA using ReverTra Ace qPCR RT Master Mix (Toyobo, Osaka, Japan). qRT-PCR was performed on CFX96 Touch Real-Time PCR Detection System (Bio-Rad, Hercules, California, USA) using advanced universal SYBR Green Supermix (Bio-Rad, Hercules, California, USA) at the following conditions: 95℃ for 30 s, 40 cycles of 95℃ for 15 s, and 60℃ for 30 s. PCR primers used are summarized in Table 1. Fold changes in the mRNA expression levels of interferon (IFN)-γ, interleukin (IL)-1β, IL-6, and tumor necrosis factor (TNF)-α were determined relative to the control glyceraldehyde 3-phosphate dehydrogenase (GAPDH) gene expression using the 2^−ΔΔ^CT method.

**Table 1:** Primer sequences for quantitative reverse-transcription polymerase chain reaction analysis

Target gene	Primer sequence (5′–3′)	Product size (bp)
IFN-γ	Forward: 5′- GGCAAAAGGACGGTAACACG-3′ Reverse:5′- TTCACCTCGAACTTGGCGAT-3′	196
IL-1β	Forward: 5′- GGCTGACAGACCCCAAAAGA-3′ Reverse:5′- TGTCGAGATGCTGCTGTGAG-3′	101
IL-6	Forward: 5′- AGAGACTTCCAGCCAGTTGC-3′ Reverse:5′- AGTCTCCTCTCCGGACTTGT-3′	85
TNF-α	Forward: 5′- CTGTGCCTCAGCCTCTTCTC-3′ Reverse:5′- ACTGATGAGAGGGAGCCCAT-3′	126
GAPDH	Forward: 5′- GCGAGATCCCGCTAACATCA-3′ Reverse:5′- CTCGTGGTTCACACCCATCA-3′	178

### Oil Red O staining

The SS was harvested from five specimens of each group, after which the muscular portion was meticulously dissected. Each sample was frozen at − 80 °C and cut to frozen tissue sections at a thickness of 8 mm for Oil Red O staining (MAK194, Sigma-Aldrich, St. Louis, Missouri, USA) and counterstaining with hematoxylin [1, 26, 27]. Oil Red O staining is used for the histologic visualization of fat cells and neutral fat. The FI of specimens was evaluated under a light microscope and with ImageJ.

### Histological evaluation

The RC SS muscles of the experimental and control groups were harvested. Harvested samples were fixed in neutral buffered 10% formalin (pH 7.4), and paraffin blocks were fabricated [27]. The paraffin blocks with a thickness of 4-lm were deparaffinized and rehydrated. Second, sample slides were randomly selected and stained with hematoxylin and eosin, Masson’s trichrome, and picrosirius red. Analyses were performed by two investigators who were blinded to the animal groups. Each tissue slide was imaged under a microscope (Leica DM IL LED; Leica Microsystems, Wetzlar, Germany) using LAS V4.8 (Leica Microsystems) imaging system [1]. To quantify changes in muscle fiber area, H&E images were analyzed using ImageJ software (Public domain JAVA image processing program, National Institute of Health, Bethesda, Maryland, USA).

### Statistical analysis

Statistical analysis was performed using SPSS Statistics for Windows version 12.0 (SPSS Inc., Chicago, IL, USA). An independent t test was performed to evaluate the comparison of proinflammatory cytokine expression and quantitative comparison of FI of the SS between the two groups. Data are presented as mean and standard deviation (SD). Statistical significance was set at *P* < 0.05.

## Results

### In acute RC tendon rupture, metformin increases the expression of proinflammatory cytokines

**Fig. 3:**
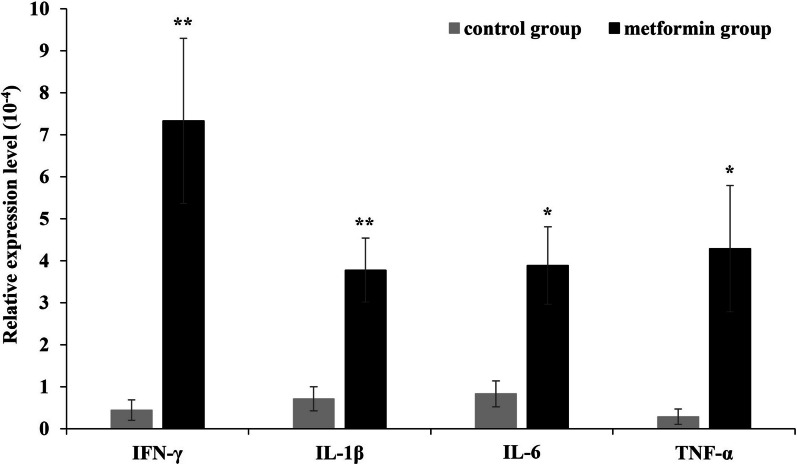
Expression level of proinflammatory cytokines on the SS treated with metformin. In this graph, proinflammatory cytokines interferon gamma (7.2-fold, *P* < .01), tumor necrosis factor alpha (13-fold, *P* < .05), interleukin 1-beta (4.7-fold, *P* < .05), and interleukin-6 (4.6-fold, *P* < .01) in the metformin group were significantly increased compared with those in the control group. The values are presented as means, with the error bars depicting the standard error of the mean (*n* = 9). *P < .05; **P < .01; ***P < .001

To confirm the inflammation-modulating action of metformin in RC rupture, changes in the expression of proinflammatory cytokines were confirmed. The SS muscle sampling point was 5 days, when the inflammatory response peaked after RC rupture, which was confirmed in a previous study. As shown in Fig. 3, proinflammatory cytokines IFN-γ (7.2-fold, *P* < .01), TNF-α (13-fold, *P* < .05), IL1β (4.7-fold, *P* < .05), and IL6 (4.6-fold, *P* < .01) in the metformin group were significantly increased compared with those in the control group (Fig. 3).

### In the presence of RC tendon rupture, metformin inhibits SS FI

**Fig. 4:**
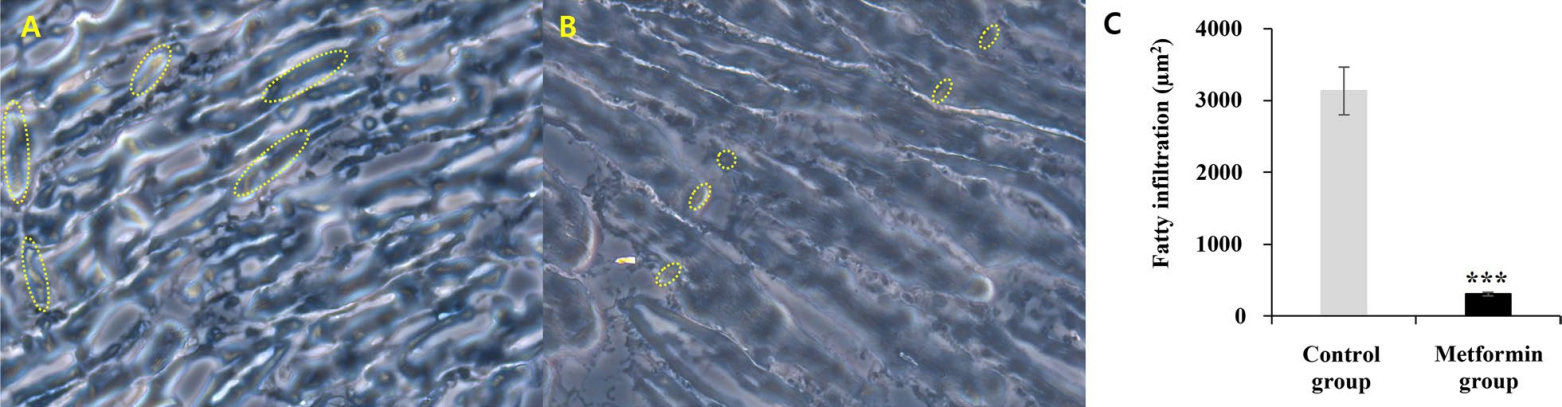
Oil Red O staining of the metformin group **A** and the control group **B** 14 days after surgery. The spot (yellow arrows) of orange represents FI in the SS after tendon tear (magnification, ×50). The metformin group **B** showed a significantly lower FI than the control group. In graph **C**, the FI of the metformin group (305 ± 50.3 µm^2^) was suppressed by nearly 10 times compared with that in the control group (3135.6 ± 662.1 µm^2^). The values are presented as means, with the error bars represent the standard deviation of the mean (*n* = 5). ***P < .001

Oil Red O staining was performed to evaluate metformin’s inhibitory effect of SS FI after RC rupture. As shown in Fig. 4, the metformin group had significantly lower FI than the control group (Fig. 4A, B). In the quantitative analysis, results confirmed that the FI of the metformin group (305 ± 50.3 µm^2^, *P* < .001) was suppressed by nearly 10 times compared with that of the control group (3135.6 ± 662.1 µm^2^, *P* < .001) (Fig. 4C). These results demonstrate that the use of metformin in the acute inflammatory phase after RC rupture can inhibit SS FI.

### Metformin prevents SS atrophy and structural damage to muscle fibers

**Fig. 5:**
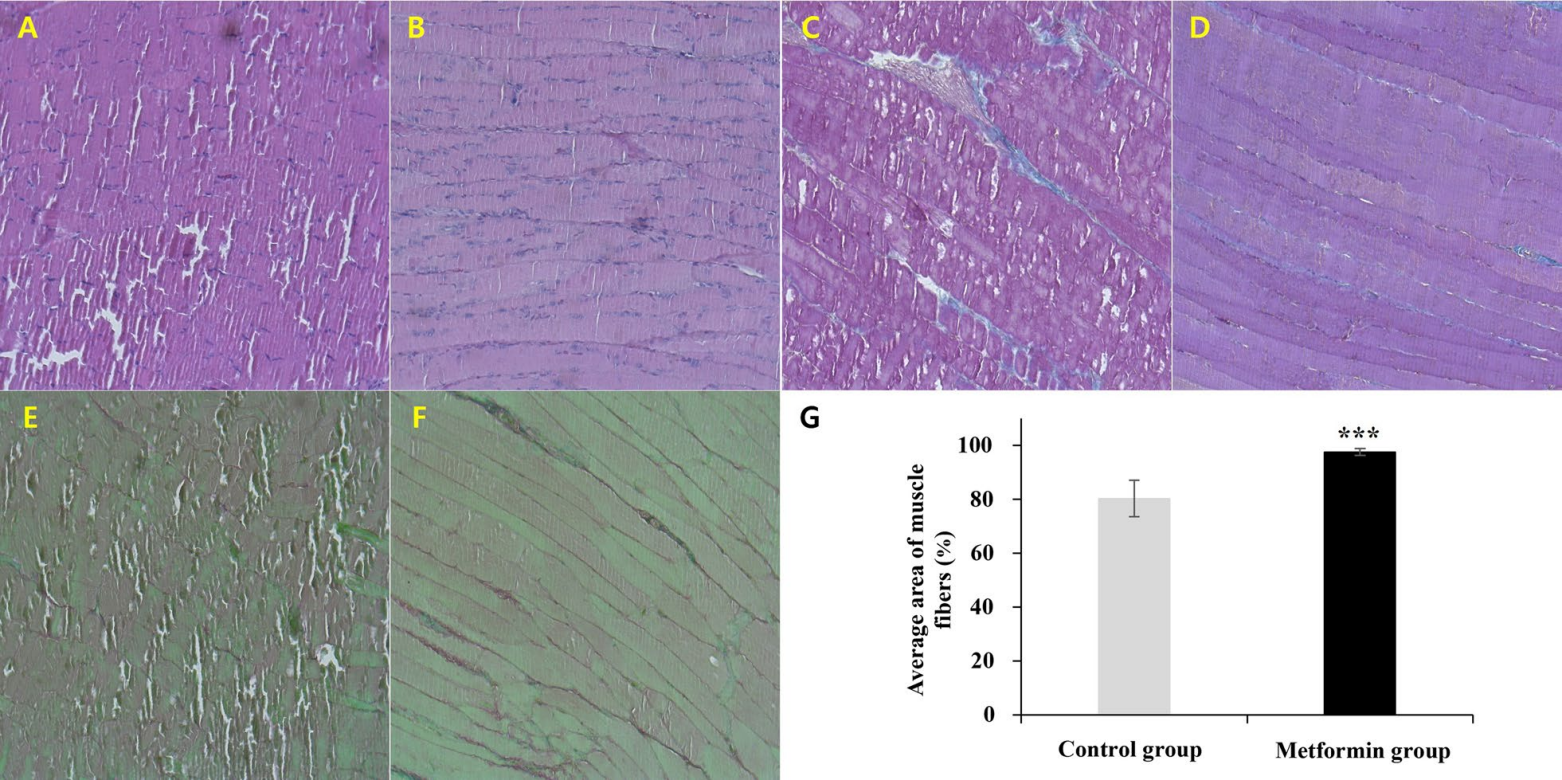
Effect of metformin on morphological changes in the SS muscle. Metformin group: **B**, **D**, and **E**. Control group: **A**, **C**, and **E**. **A**, **B** Hematoxylin and eosin. **C**, **D** Masson’s trichrome. **E–F** Picrosirius red staining. **G** represents the average area of muscle fiber measured by ImageJ software. The values are presented as means, with the error bars depicting the standard deviation of the mean (*n* = 10). ***P < .001. All images by ×100 magnification

Through histological macroscopic analysis, structural changes were observed in the SS muscle tissue of the metformin group. As shown in Fig. 5, muscle fibers from rats with delayed suturing after RC rupture showed morphological changes such as atrophy, heterogeneous arrangement, and wide interfibrillar voids. Overall, myofibrillar structure and morphology were disrupted by muscle atrophy (Fig. 5A, C, E). By contrast, the muscle fibers of metformin-treated rats after RC rupture showed a homogeneous arrangement of collagen fibers and narrow interfiber voids (Fig. 5B, D, F). In the quantification of the atrophied muscle, the control group (80.27 ± 6.76%, *P* < .001) showed a 21.6% reduction in muscle fiber area compared to the metformin group (97.60 ± 1.22%,* P* < .001) (Fig. 5G). This indicates that metformin prevented muscle tissue atrophy and myofibrillar structural damage induced in delayed repair after RC tear.

## Discussion

In this study, we confirmed metformin-induced increase in proinflammatory cytokines and suppression of SS FI in acute RC tendon rupture. Proinflammatory cytokines, released by immune cells under inflammatory conditions, play a pivotal role in muscle fat degeneration [14, 28, 29]. Metformin has the potential to activate T cells and macrophages and modulate adipocyte function [30]. This study suggests the effectiveness of metformin for inhibition of SS FI induced by acute RC tear.

Metformin is known to exhibit anti-inflammatory effects by inhibiting the release of proinflammatory cytokines, which generally play an important role in the occurrence and development of inflammation [31–33]. This may differ from our results. However, this discrepancy could be explained by metformin-induced activation of inflammatory macrophages in RC tear. Intramuscular adipocytes arise primarily from the FAPs population that resides between muscle fibers. In fact, infiltrating inflammatory macrophages can directly induce apoptosis of FAPs through the secretion of TNF-a [24] and can strongly suppress the adipogenesis of FAPs by satellite cell-derived muscle fibers by secreting IL-6 and G-CSF [25]. In a previous study showed that pretreatment with metformin in concanavalin A hepatitis increased the total number of hepatic infiltrating immune cells (CD_4_^+^ T, CD8^+^ T, and NK_1.1_^+^CD3^-^ NK cells) and correspondingly increased TNF-α, IFN-γ and IL-17 [30]. In addition, it was confirmed that metformin increased proinflammatory cytokines (TNF-α, IL-1α, IL-1β, IL-6, IL-18, IL-8, and IFN-α) in *Mycobacterium tuberculosis* (causative agent of tuberculosis)-infected macrophages [34]. Overall, in the present study, the increased proinflammatory cytokines by metformin treatment in RC tear could be due to activated macrophages, and the suppression of SS FI could be due to inhibition of the differentiation ability of FAP by inflammatory macrophages or induction of apoptosis.

Although the underlying pathogenesis of RC SS FD remains unclear, most agree with the importance of immune cell infiltration [35]. In RC rupture, the SS shows severe signs of acute inflammation, premature degeneration, and FI. The rupture of the RC SS tendon increases the presence of immune cells such as leukocytes, neutrophils, and macrophages and increases the levels of proinflammatory cytokines [1, 17]. This acute inflammation peaks on days 5–7, and the SS fatty infiltrate worsen in the absence of other therapeutic interventions [5]. A large-scale RC tendon tear animal model study also reported similar or more pronounced FI results [28]. The results of this study showed that early administration of metformin in RC rupture could effectively inhibit SS FI. This is because metformin upregulates proinflammatory cytokines, and this is confirmed by our results. In addition, the regulation of cytokines by metformin prevented the decrease in the volume of SS muscle tissue caused by muscle atrophy on day 14 after surgery and prevented the disintegration of the shape and structure.

Muscular FD by RC tear cannot be reversed by suturing and may increase extramuscular fatty accumulation. In addition, since RC FD adversely affects postoperative prognosis, potential therapeutic targets must be developed [36]. Several animal studies have shown that FD and atrophy can be inhibited through the inhibition of specific genes [13, 37], application of adipose-derived stem cells [38], application of perivascular stem cells [39], or improvement of hypercholesterolemia [40]. However, data on the regulation of proinflammatory cytokines in the acute inflammatory response underlying RC muscle FD are limited. Through this study, we suggest that the therapeutic intervention of metformin, which can promote the expression of proinflammatory cytokines in the early stages of RC rupture, can effectively inhibit FI.

The effect of metformin to inhibit SS FI in RC tears was reported in this study for the first time; however, some limitations should be considered in interpreting the results. First, since the expression of time-dependent cytokines in the early stages of RC rupture is a much more dynamic phenomenon, it must be measured at more points. In addition, the expression of cytokines in cells that play important roles in the immune response, such as neutrophils and macrophages, should be directly investigated. Second, the limited evaluation period of 2 weeks does not consider all possible subsequent events and may not be sufficient for the final analysis. Third, we only assessed a singular dose of metformin, which may not adequately account for its efficacy. Therefore, future investigations should encompass the evaluation of SS FI inhibition across a minimum of two doses or even higher doses. Fourth, we did not explore the impact of FI inhibition on enhancing the biomechanical aspects of tendon healing following tendon repair. Subsequent research endeavors should validate the potential biomechanical enhancements in tendon healing attributed to metformin, utilizing an acute RC repair rat model. Fifth, the histological evaluation was semi-quantitative, and a histological system specific to RC tears was not used. Finally, this study successfully confirmed the expression of proinflammatory cytokines. Nonetheless, for a thorough assessment of FI inhibition, it is necessary to conduct molecular marker (PPAR-γ and C/EBP-α) analysis and include immunohistochemical staining experiments.

In conclusion, our study demonstrates that the initial use of metformin promotes the expression of proinflammatory cytokines in an acute RC rupture rat model. SS FI promoted by delayed RC tear suturing could be effectively inhibited by metformin. In addition, metformin can preserve the morphology and structure of the SS muscle following muscle atrophy. Therefore, metformin can be an effective treatment to inhibit muscle atrophy and FI following RC tears.

## Conclusions

In acute RC rupture, the use of metformin may increase the levels of proinflammatory cytokines, which are mediators of inflammatory response. The control of this acute inflammatory response could be a new therapeutic strategy to prevent muscle FD. Metformin promotes the inflammatory response in the early stages of RC rupture and can effectively prevent muscle atrophy and FI.

## Data Availability

The datasets used and/or analyzed during the current study are available from the corresponding author on reasonable request.

## Abbreviations

RC: Rotator cuff
SS: Supraspinatus
FI: Fatty infiltration
FD: Fatty degeneration
IFN-γ: Interferon-gamma
IL-1β: Interleukin-1 beta
IL-6: Interleukin-6
TNF-α: Tumor necrosis factor-alpha
GAPDH: Glyceraldehyde 3-phosphate dehydrogenase
qRT-PCR: Quantitative reverse-transcription polymerase chain reaction
SD: Standard deviation

## References

[1] Yoon JP, Min SG, Choi JH, Lee HJ, Park KH, Yoon SH (2022). Increased interleukin-6 and TP53 levels in rotator cuff tendon repair patients with hypercholesterolemia. Clin Shoulder Elbow.

[2] Blaine TA, Kim Y-S, Voloshin I, Chen D, Murakami K, Chang S-S (2005). The molecular pathophysiology of subacromial bursitis in rotator cuff disease. J Shoulder Elbow Surg.

[3] Harryman DT, Hettrich CM, Smith KL, Campbell B, Sidles JA, Matsen FA (2003). A prospective multipractice investigation of patients with full-thickness rotator cuff tears: the importance of comorbidities, practice, and other covariables on self-assessed shoulder function and health status. J Bone Joint Surg.

[4] Sakai H, Fujita K, Sakai Y, Mizuno K (2001). Immunolocalization of cytokines and growth factors in subacromial bursa of rotator cuff tear patients. Kobe J Med Sci.

[5] Shindle MK, Chen CC, Robertson C, DiTullio AE, Paulus MC, Clinton CM (2011). Full-thickness supraspinatus tears are associated with more synovial inflammation and tissue degeneration than partial-thickness tears. J Shoulder Elbow Surg.

[6] Shinohara I, Kataoka T, Mifune Y, Inui A, Sakata R, Nishimoto H (2022). Influence of adiponectin and inflammatory cytokines in fatty degenerative atrophic muscle. Sci Rep.

[7] Gladstone JN, Bishop JY, Lo IK, Flatow EL (2007). Fatty infiltration and atrophy of the rotator cuff do not improve after rotator cuff repair and correlate with poor functional outcome. Am J Sports Med.

[8] Chalmers PN, Joyce CD (2023). Editorial Commentary: Rotator cuff repair in patients with diabetes: stricter glycemic control for the first 6 months is associated with better healing. Arthroscopy.

[9] Lee YS, Jeong JY, Park C-D, Kang SG, Yoo JC (2017). Evaluation of the risk factors for a rotator cuff retear after repair surgery. Am J Sports Med.

[10] Tashjian RZ, Hollins AM, Kim H-M, Teefey SA, Middleton WD, Steger-May K (2010). factors affecting healing rates after arthroscopic double-row rotator cuff repair. Am J Sports Med.

[11] Lee S, Park I, Lee HA, Shin SJ (2020). Factors related to symptomatic failed rotator cuff repair leading to revision surgeries after primary arthroscopic surgery. Arthroscopy.

[12] Stengaard K, Hejbøl EK, Jensen PT, Degn M, Ta TML, Stensballe A (2022). Early stage inflammation changes in supraspinatus muscle after rotator cuff tear. J Shoulder Elbow Surg.

[13] Kuenzler MB, Nuss K, Karol A, Schär MO, Hottiger M, Raniga S (2017). Neer award 2016: reduced muscle degeneration and decreased fatty infiltration after rotator cuff tear in a poly (ADP-ribose) polymerase 1 (PARP-1) knock-out mouse model. J Shoulder Elbow Surg.

[14] Hamrick MW, McGee-Lawrence ME, Frechette DM (2016). Fatty infiltration of skeletal muscle: mechanisms and comparisons with bone marrow adiposity. Front Endo Crinol.

[15] Steinbacher P, Tauber M, Kogler S, Stoiber W, Resch H, Sänger AM (2010). Effects of rotator cuff ruptures on the cellular and intracellular composition of the human supraspinatus muscle. Tissue Cell.

[16] Frich LH, Fernandes LR, Schrøder HD, Hejbøl EK, Nielsen PV, Jørgensen PH (2021). The inflammatory response of the supraspinatus muscle in rotator cuff tear conditions. J Shoulder Elbow Surg.

[17] Rubino LJ, Stills HF, Sprott DC, Crosby LA (2007). Fatty infiltration of the torn rotator cuff worsens over time in a rabbit model. J Arthrosc Relat Surg.

[18] Zhang J, Brown R, Hogan MV, Onishi K, Wang JHC (2022). Metformin improves tendon degeneration by blocking translocation of HMGB1 and suppressing tendon inflammation and senescence in aging mice. J Orthop Res.

[19] Evans JM, Donnelly LA, Emslie-Smith AM, Alessi DR, Morris AD (2005). Metformin and reduced risk of cancer in diabetic patients. Brit Med J.

[20] Feng YY, Wang Z, Pang H (2022). Role of metformin in inflammation. Mol Biol Rep.

[21] Holman RR, Paul SK, Bethel MA, Matthews DR, Neil HAW (2008). 10-year follow-up of intensive glucose control in type 2 diabetes. N Engl J Med.

[22] Salvatore T, Pafundi PC, Galiero R, Rinaldi L, Caturano A, Vetrano E (2021). Can metformin exert as an active drug on endothelial dysfunction in diabetic subjects?. Biomedicines.

[23] Salvatore T, Galiero R, Caturano A, Vetrano E, Rinaldi L, Coviello F (2021). Effects of metformin in heart failure: from pathophysiological rationale to clinical evidence. Biomolecules.

[24] Moratal C, Raffort J, Arrighi N, Rekima S, Schaub S, Dechesne CA (2018). IL-1β- and IL-4-polarized macrophages have opposite effects on adipogenesis of intramuscular fibro-adipogenic progenitors in humans. Sci Rep.

[25] Uezumi A, Fukada S-i, Yamamoto N, Si T, Tsuchida K (2010). Mesenchymal progenitors distinct from satellite cells contribute to ectopic fat cell formation in skeletal muscle. Nat Cell Biol.

[26] Kim DH, Min SG, Kim H-M, Kang HR, Choi J-H, Lee HJ (2022). Comparison of the characteristics of rotator cuff tissue in a diabetic rat model. Orthopedics.

[27] Yoon JP, Kim DH, Min SG, Kim HM, Choi JH, Lee HJ (2022). Effects of a graphene oxide-alginate sheet scaffold on rotator cuff tendon healing in a rat model. J Orthop Surg.

[28] Kuzel BR, Grindel S, Papandrea R, Ziegler D (2013). Fatty infiltration and rotator cuff atrophy. J Am Acad Orthop Surg.

[29] Aaron N, Zahr T, He Y, Yu L, Mayfield B, Pajvani UB (2022). Acetylation of PPARγ in macrophages promotes visceral fat degeneration in obesity. Life Metab.

[30] Volarevic V, Misirkic M, Vucicevic L, Paunovic V, Simovic Markovic B, Stojanovic M (2015). Metformin aggravates immune-mediated liver injury in mice. Arch Toxicol.

[31] Saffari PM, Alijanpour S, Takzaree N, Sahebgharani M, Etemad-Moghadam S, Noorbakhsh F (2020). Metformin loaded phosphatidylserine nanoliposomes improve memory deficit and reduce neuroinflammation in streptozotocin-induced alzheimer's disease model. Life Sci.

[32] Qi B, Hu L, Zhu L, Shang L, Wang X, Liu N (2017). Metformin attenuates neurological deficit after intracerebral hemorrhage by inhibiting apoptosis, oxidative stress and neuroinflammation in rats. Neurochem Res.

[33] Vasamsetti SB, Karnewar S, Kanugula AK, Thatipalli AR, Kumar JM, Kotamraju S (2015). Metformin inhibits monocyte-to-macrophage differentiation via AMPK-mediated inhibition of STAT3 activation: potential role in atherosclerosis. Diabetes.

[34] Naicker N, Rodel H, Perumal R, Ganga Y, Bernstein M, Benede N (2023). Metformin increases cell viability and regulates pro-inflammatory response to Mtb. Infect Drug Resist.

[35] Gumucio J, Flood M, Harning J, Phan A, Roche S, Lynch E (2014). T lymphocytes are not required for the development of fatty degeneration after rotator cuff tear. Bone Joint Res.

[36] Fuchs B, Weishaupt D, Zanetti M, Hodler J, Gerber C (1999). Fatty degeneration of the muscles of the rotator cuff: assessment by computed tomography versus magnetic resonance imaging. J Shoulder Elbow Surg.

[37] Davies MR, Liu X, Lee L, Laron D, Ning AY, Kim HT (2016). TGF-β small molecule inhibitor SB431542 reduces rotator cuff muscle fibrosis and fatty infiltration by promoting fibro/adipogenic progenitor apoptosis. PLoS ONE.

[38] Oh JH, Chung SW, Kim SH, Chung JY, Kim JY (2014). 2013 Neer award: effect of the adipose-derived stem cell for the improvement of fatty degeneration and rotator cuff healing in rabbit model. J Shoulder Elbow Surg.

[39] Eliasberg CD, Dar A, Jensen AR, Murray IR, Hardy WR, Kowalski TJ (2017). Perivascular stem cells diminish muscle atrophy following massive rotator cuff tears in a small animal model. J Bone Joint Surg Am.

[40] Klomps LV, Zomorodi N, Kim HM (2017). Role of transplanted bone marrow cells in development of rotator cuff muscle fatty degeneration in mice. J Shoulder Elbow Surg.

